# A Novel Nanoprobe Based on Core–Shell Au@Pt@Mesoporous SiO_2_ Nanozyme With Enhanced Activity and Stability for Mumps Virus Diagnosis

**DOI:** 10.3389/fchem.2020.00463

**Published:** 2020-06-05

**Authors:** Lin Long, Rui Cai, Jianbo Liu, Xiaochun Wu

**Affiliations:** ^1^College of Opto-electronic Engineering, Zaozhuang University, Zaozhuang, China; ^2^Zaozhuang Municipal Center for Disease Control and Prevention, Zaozhuang, China; ^3^CAS Key Laboratory of Standardization and Measurement for Nanotechnology, CAS Center for Excellence in Nanoscience, National Center for Nanoscience and Technology, Beijing, China; ^4^University of Chinese Academy of Sciences, Beijing, China

**Keywords:** Au nanorods, platinum, mesoporous SiO_2_, core–shell, nanozyme, enhanced activity, virus diagnosis

## Abstract

Nanoporous materials with core-shell structure have received lots of attention owing to the great versatility of the functional cores and shells and their potential application in catalysis and biological applications. In this work, a facile method has been developed to synthesize uniform Au@Pt@mesoporous SiO_2_ nanostructures with high peroxidase-like activity, which had a well-defined core–shell structure with Au nanorods@Pt nanodots as a core and mesoporous SiO_2_ as a shell. The mesoporous SiO_2_ shell can not only provide convenient transmission channels but offer a substantial location for accommodation of large biomolecules, such as antibodies and antigens. Here a novel nanoprobe based on Au@Pt@mesoporous SiO_2_ nanozyme modified with mumps antigens was reported. Notably, the encapsulation of Au@Pt nanorod in mesoporous SiO_2_ shell was able to hinder the interaction between catalytical nanoparticles and recognition antigens, retaining the catalytic activity of the inner active nanoparticle core. Furthermore, this nanoprobe exhibited an extraordinarily stability and showed excellent activity. As a result, we presented an enzyme linked immunosorbant assay (ELISA) for the diagnosis of mumps virus; this proposed method exhibited good sensitivity to mumps-specific IgM antibodies. The limit of detection can be as low as 10 ng/mL, which was more sensitive compared to the conventional immunoassay. Our results indicated that this nanoprobe hold great promise with opportunities for applications of biosensors, catalysis and biotechnology.

## Introduction

Mumps is a common childhood infectious disease caused by a the mumps virus. Although most cases of infection lead to a mild disease, orchitis, permanent deafness, and disability are some untoward effects of mumps (Galazka et al., 1999). A laboratory diagnosis of mumps is based on detection of viral nucleic acid using polymerase chain reaction (PCR), isolation of the virus from saliva or spinal fluid, or serological confirmation (Maillet et al., 2015). In the absence of successful virus isolation in cell-culture or reverse transcription-PCR (RT-PCR) detection, serological markers can provide a simple, and useful diagnosis (Hviid et al., 2008). A commonly used serological confirmation for the rapid diagnosis of mumps infection is demonstration of specific immunoglobulin M (IgM) class antibody (Krause et al., 2007). However, mumps-specific IgM antibodies might be low or undetectable if serum are collected before 4 days of clinical presentation, thus giving false-negative results (Warrener and Samuel, 2006). For this reason, a simple and sensitive laboratory diagnosis of mumps virus is needed. Among various efficient bioassays for diagnosing infectious diseases, natural enzyme labels have shown great potential in various bioassays, as they can catalyze various colorimetric reactions with good sensitivity and selectivity toward the target molecules (Gut et al., 1985). However, methods based on enzyme labels also have several limitations including the natural instability of proteins during long-term operation or storage (Rashidian et al., 2013). Nanozyme with catalytical-like activity have been emerging alternatives to natural enzymes in bioassays (Gao et al., 2007). The replacement of natural enzymes by catalytical nanomaterials in immunoassay may have advantages in several aspects, such as their shape- (structure-, size-, composition-) tunable catalytic activities, large surface area for bioconjugation and modification, greater resistance to extremes of pH and temperature, and so on (Wu et al., 2019).

**Figure 1:**
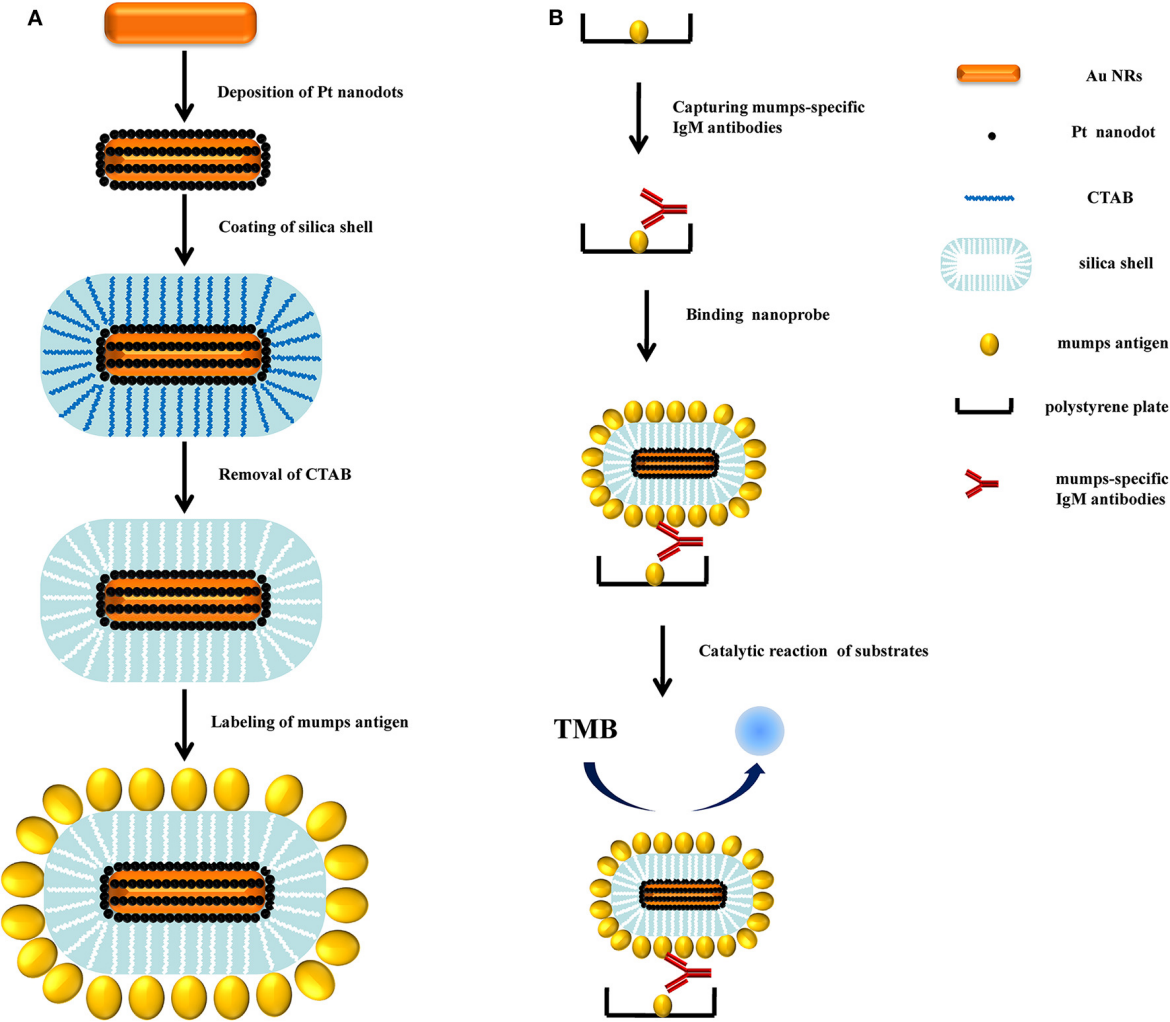
**(A)** Schematic diagram of the fabrication procedure for the Ags-APMSN. **(B)** Schematic illustration of the immunoassay of Ags-APMSN based ELISA system.

Recently Pt nanoparticles have been found to exhibit catalase-like, superoxide dismutase (SOD)-like, peroxidase-like, and oxidase-like activities (Ma et al., 2011). Considering the size- and shape-dependent properties, nanosized Pt nanoparticles of 2~4 nm diameter are found to have the excellent catalytic activity. However, small nanoparticles are unstable and tend to agglomerate to larger clusters, resulting in the loss of their original catalytic activity (Narayanan and El-Sayed, 2004). Various efforts have been made to fabricate novel nanostructures with higher activity and lower cost for catalytic applications (Zhang et al., 2007). In particular, gold nanoparticle has been regarded as a well-known bio-materials for their excellent biocompatibility and large specific surface area; thus, Pt covering Au or Au-Pt core–shell nanoparticle structure have been reported for a wide range of applications (Wu et al., 2018). Previously, our group found Au@Pt core/shell nanostructures exhibit peroxidase activity and such a structure was highly desirable for catalysis, since the large surface area of gold nanorod (NR) provided numerous adhesion sites for the small Pt nanodots (Liu et al., 2012). With this in mind, herein, we developed a facile method to fabricate a new nanozyme by encapsulating Au@Pt NR in mesoporous SiO_2_ shell (Figure 1A). The obvious advantages of mesoporous SiO_2_ shell are the high surface area, open mesoporous channel and easy modified properties (Lin et al., 2013). On the basis of these characteristics, mesoporous SiO_2_-coated nanoparticles are proposed as an ideal carrier for loading biomolecules (Zhang et al., 2012). Thus, in this study, a novel nanoprobe for mumps virus serodiagnosis was developed by modifying the Au@Pt@mesoporous SiO_2_ nanozyme (APMSN) with mumps antigens. Using captured-type ELISA, we demonstrated the applicability of this antigens-conjugated APMSN (Ags-APMSN) for the reliable, simple, and sensitive detection of mumps-specific IgM antibodies (Figure 1B).

## Materials and Methods

### Materials, Reagents, and Instruments

Cetylmethylammonium bromide (CTAB), sodium borohydride (NaBH_4_), chloroauric acid (HAuCl_4_·3H_2_O), silver nitrate (AgNO_3_), potassium tetrachloroplatinate (II) (K_2_PtCl_4_), L-ascorbic acid (AA), tetraethyl orthosilicate (TEOS), Sodium hydroxide (NaOH), sulfuric acid (H_2_SO_4_), hydrogen peroxide (H_2_O_2_), 2,2′-azino-bis(3-ethylbenzo-thiazoline-6-sulfonic acid) diammonium salt (ABTS), 3,3′,5,5′-tetramethylbenzidine (TMB) and o-phenylenediamine (OPD) were purchased from Alfa Aesar (USA). Mumps antigen was purchased from Beijing Cy-tech Biotech Co., Ltd. (China). Mumps antigen coated plate, mumps virus IgM ELISA kit were purchased from IBL International GmbH (Germany). Transmission electron microscopy (TEM) images were acquired with Tecnai G2 T20 S-TWIN microscope. Energy dispersive X-ray analysis (EDX) and scanning transmission electron microscopy (STEM) element mappings were performed on Tecnai G2 F20 U-Twin microscope. The effective diameter and zeta potential were obtained from Zetasizer Nano ZS. UV-vis-NIR absorption spectra were obtained from Perkine-Elmer Lambda 950 and Varian Cary 50. The ELISA test was performed on Infinite™ M200.

### Preparation of Au NRs and Au@Pt NRs

A seed-mediated growth method was used to synthesize Au NRs. First, Au seeds were synthesized by reduction of HAuCl_4_ with NaBH_4_. 100 μL of 24 mM HAuCl_4_ was mixed with 7.5 mL of 0.1 M CTAB and 9.4 mL water. 600 μL of 0.01 M NaBH_4_ was then added. Vigorous stirring of the seed solution was continued for 3 min, the seed solution was kept undisturbed at 30°C for 30 min prior to any further experimentation. Growth solution consisted of 2.04 mL of 24 mM HAuCl_4_, 1.05 mL of 10 mM AgNO_3_, 100 mL of 0.1 M CTAB, and 2 mL of 0.5 M H_2_SO_4_. 120 μL seed solution was added to the growth solution and 800 μL of 0.1 M AA to initiate the growth of Au NRs. After 12 h, the nanorods can be concentrated by centrifugation at 12000 rpm for 5 min twice.

Au NR colloids (0.1 mL) were added into the mixture of K_2_PtCl_4_ (75 μL, 2 mM) and freshly prepared ascorbic acid (15 μL, 0.1 M AA). After stirring for 30 min, the obtained Au@Pt NRs can be concentrated by centrifuging at 12,000 rpm for 5 min twice.

### Preparation of APMSNs and Ags-APMSNs

Au@Pt NR solutions (10 mL) were added into the mixture of CTAB (75 μL, 0.1 M) and NaOH (50 μL, 0.2 M) with stirring. Three 30 μL TEOS (20%) were added at 30 min intervals. The mixture was stirring for 24 h in a 30°C water bath. The obtained APMSNs can be concentrated by centrifuging at 12,000 rpm for 5 min twice. To remove the CTAB template, the precipitate was collected and re-dispersed into 60 mL ethanol/NH_4_NO_3_ solution (6 g/L) for 24 h at 50°C, and then centrifuged at 12,000 rpm for 5 min twice with ethanol.

APMSNs solution (50 μL 5 nM) were added into the mixture of PBS buffer (900 μL, 0.1 M, pH 7.4) and mumps antigen (50 μL, 10 mg/mL) and incubated for 96 h at 4°C. The obtained Ags-APMSNs were can be concentrated by centrifuging at 12,000 rpm for 5 min twice. The precipitate was re-dispersed in PBS buffer (100 μL, 0.1 M, pH 7.4).

### Measurement of Peroxidase-Like Activity of Ags-APMSNs

Experiments on the TMB-H_2_O_2_ catalytic reaction were carried out with 0.4 mM TMB, 100 mM H_2_O_2_, and 0.05 nM Ags-APMSNs in a reaction volume of 3 mL. Unless otherwise stated, the reaction was carried out at 30°C in PBS buffer (0.1 M, pH 5) and used for absorption spectroscopic measurements at 650 nm.

### Detection of Mumps-Specific IgM Antibodies by ELISA

Firstly, 100 μL per well of mumps antigen was loaded into the wells of a 96-well microtiter plate for 12 h at 4°C. The plates were then washed three times with PBST buffer. After that, 200 μL of 5% BSA in PBS was added to the each wells and incubated for 3 h at 30°C. After the wells were washed with PBST buffer three times, 100 μl of diluted samples were added to the each wells and incubated at 37°C for 0.5 h. After the wells were washed with PBST buffer three times, 100 μl Ags-APMSNs were added to wells and incubated at 37°C for 0.5 h. After the wells were washed with PBST buffer three times, 100 μL PBS buffer (pH 5) of substrate solution containing 0.4 mM TMB and 100 mM H_2_O_2_ was added into each well. After incubating at 30°C for 10 min, the absorbance of each well was measured at 650 nm by a microplate reader.

## Results and Discussion

### Characterization of APMSNs and Ags-APMSNs

**Figure 2:**
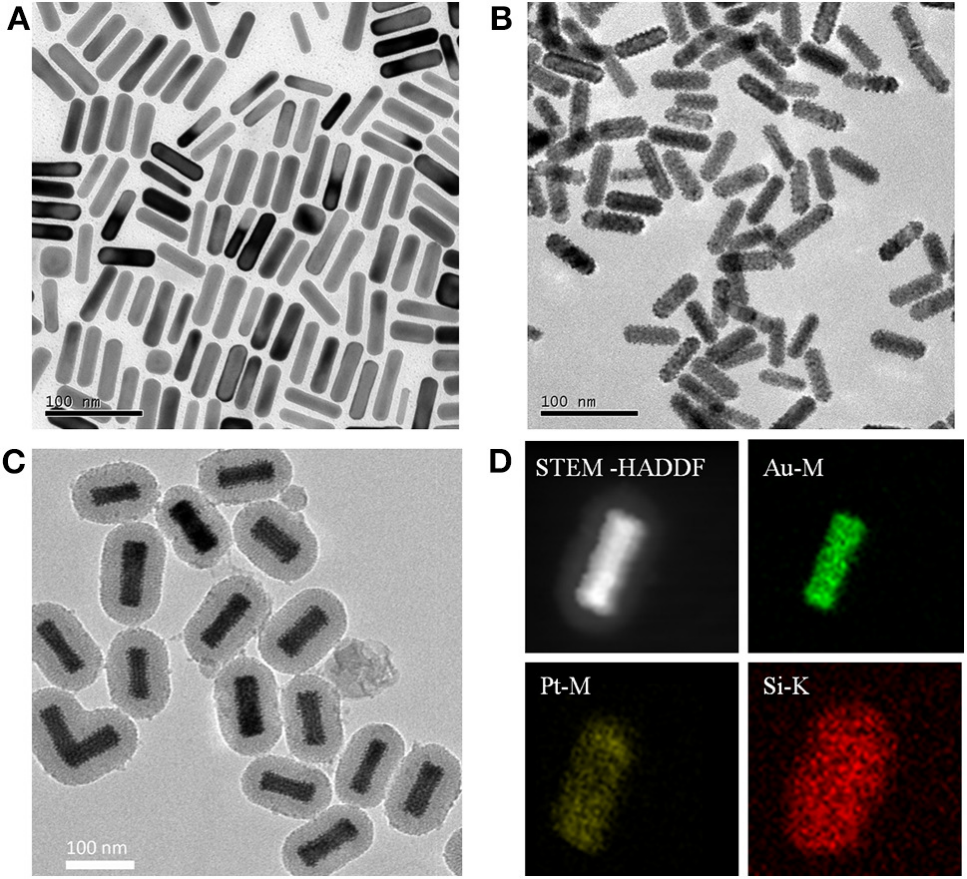
Typical TEM images of **(A)** Au NRs, **(B)** Au@Pt NRs, and **(C)** APMSNs. **(D)** STEM-HAADF image and STEM-EDX maps of Au, Pt, and Si, respectively.

The working principle of the rationally designed APMSNs was schematically represented in Figure 1A. Au NRs (Figure 2A) were used as seeds to for subsequent overgrowth of Pt nanodots. Pt nanodots with sizes of 2–3 nm formed a nanoisland shell on Au NRs from the transmission electron microscopy (TEM) image (Figure 2B). Obviously, the Au NRs provided a well-dispersed surface distribution of Pt nanodots. The surfaces of Au@Pt NRs are stabilized by the CTAB surfactants. The CTAB molecules, can also serve as template for the formation of the mesoporous silica shell. The average thickness of the mesoporous SiO_2_ layer surrounding the Au@Pt NR was around 25 nm (Figure 2C). Figure 2D showed STEM images and EDX element mappings of Au, Pt, and Si for one selected nanoparticle. Pt are found in the shell outside the Au core, and Si are in the outer shell. Furthermore, after the removal of the CTAB by NH_4_NO_3_/ethanol solution, the channel-like mesopores were all open and gave good mass transport and accessibility to their internal surfaces.

**Figure 3:**
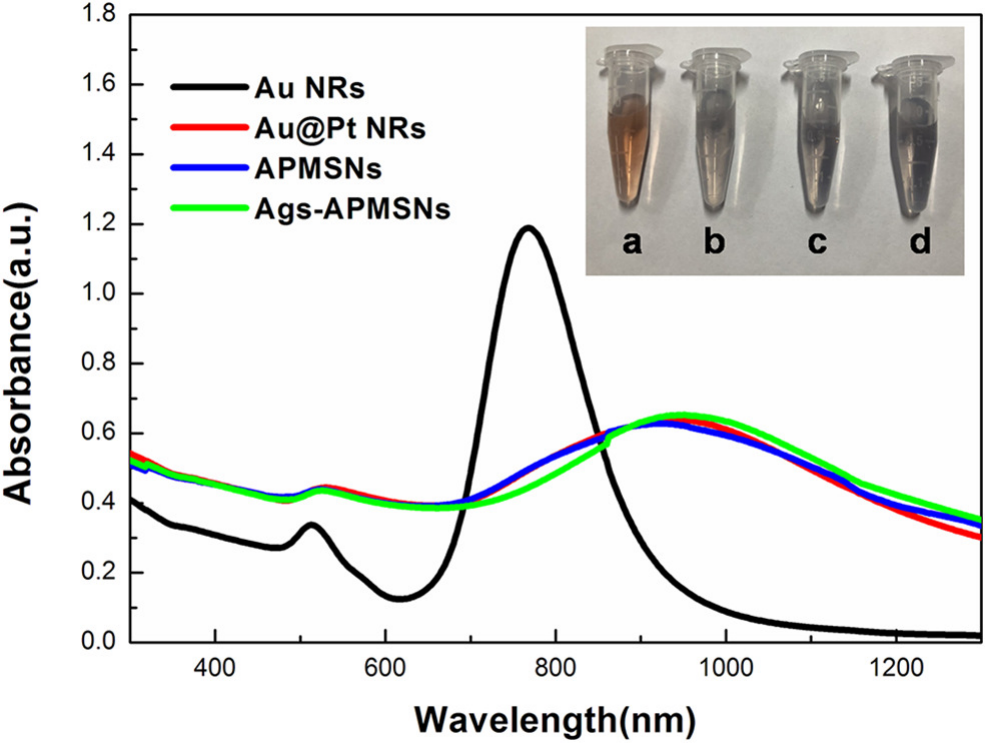
UV-vis absorption spectra of Au NRs, Au@Pt NRs, APMSNs and Ags-APMSNs. Insets are the corresponding photographs of the **(a)** Au NRs, **(b)** Au@Pt NRs, **(c)** APMSNs, and **(d)** Ags-APMSNs solutions.

Due to the phenomenon of surface plasmon resonance (SPR), the Au NRs exhibit striking optical properties. The SPR properties of Au NRs depend on the aspect ratio and the dielectric constant of gold and surrounding medium. In our experiment, Au NRs exhibited a longitudinal SPR (LSPR) peak at 768 nm (Figure 3), and the LSPR peak of the Au@Pt NRs shifted toward the red direction (892 nm) when Pt nanodots were formed on the surface of Au NRs. The quite large red-shift and intensity damping here mainly came from the dielectric constant of the Pt and the porous shell structure of the Pt nanodots. Figure 3 also showed that upon surface modification by mesoporous SiO_2_ layer and antigen molecular, the position or width of the Au@Pt NRs remained almost unchanged.

In this study, we used dynamic light scattering (DLS) measurements to determine the surface potential of the nanoparticles (Table 1). The positive charge (ζ = +20 mV) developed in Au NRs and Au@Pt NRs were assumed to CTAB bilayer at the NR surface. As shown in Table 1, mesoporous SiO_2_ coating reversed the surface charge of the Au@Pt NRs to negative. After removal of the CTAB template there is a small loss of charge. Table 1 also illustrated that the surface potential become less negative after antigen conjugation process, due to the electrostatic interaction between the positively charged antigen and the negatively charged nanorod surface.

**Table 1:** Different characterization of five kinds of nanoparticles.

**Material**	**LSPR peak (nm)**	**Effective diameter (nm)**	**Zeta potential (mV)**
AuNRs	768	17.1 ± 0.6	20.9 ± 0.4
Au@Pt NRs	892	46.0 ± 0.5	20.8 ± 0.6
Au@Pt@ mesoporous SiO_2_ NRs with CTAB template	916	100.6 ± 0.7	−25.2 ± 0.6
APMSNs	926	92.1 ± 0.6	−19.1 ± 0.6
Ags-APMSNs	950	126.9 ± 1.5	−14.3 ± 0.5

Additionally, DLS measurements were used to monitor the effective diameter of the nanoparticles. It is worth notioning that the DLS measurements only provides an average spherical diameter; hence, due to the rod shape, the effective sizes determined by DLS measurements here cannot be taken literally. The effective sizes in our case was used to demonstrate the relative size upon the variation of coatings. The DLS results revealed that the effective diameter of Au NRs, Au@Pt NRs, and APMSNs with CTAB template were 17.1 ± 0.6, 46.0 ± 0.5, and 100.6 ± 0.7 nm, respectively. Table 1 further showed that the effective diameter of the APMSNs increased evidently from 92.1 to 126.9 nm after antigen conjugation process. These results demonstrated the successful preparation of the Ags-APMSN based on the chemical modification that can be used in the following immunoassay.

**Figure 4:**
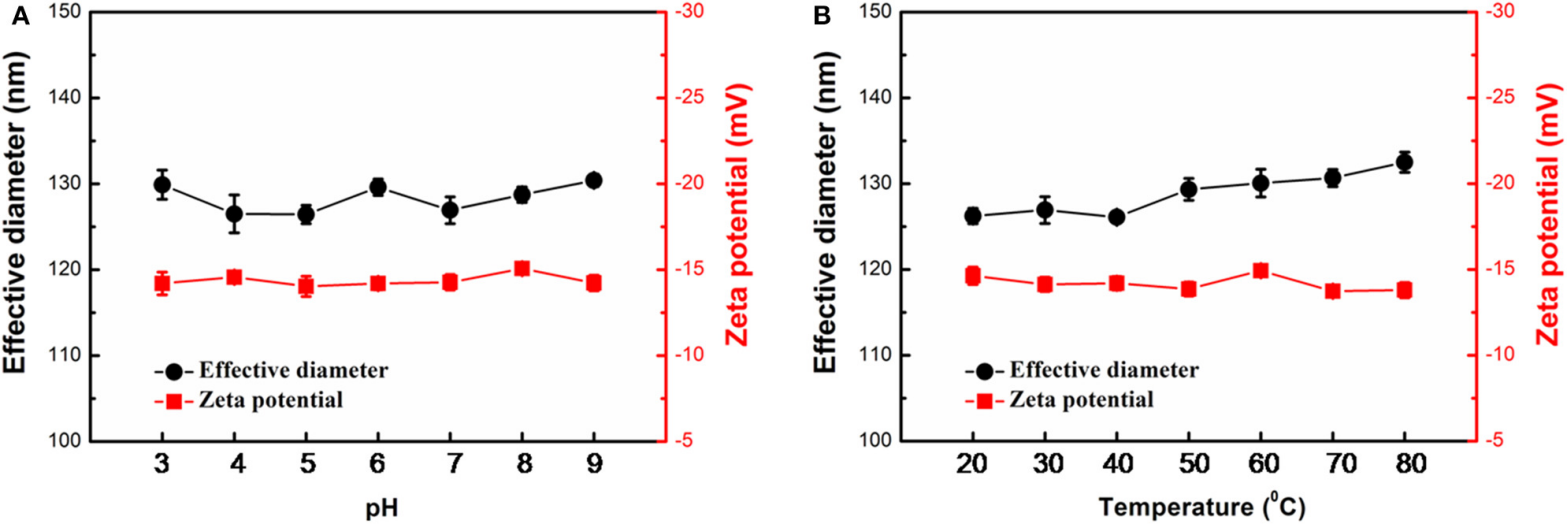
Stability of the Ags-APMSNs against pH **(A)** and temperature **(B)** variations from the viewpoints of effective diameter and Zeta potential. The samples were treated with 0.1 M PBS buffers (0.1 M) with different pH values for 3 h or incubated at different temperatures for 3 h before characterizations.

The stability of Ags-APMSNs upon pH and temperature changes were determined using DLS analysis as well. Previous studies suggested that the CTAB-stabilized Au@Pt NRs showed low dispersion stability in PBS buffers (Liu et al., 2012), whereas mesoporous shell could keep these active Au@Pt NRs catalysts robust to harsh environments (Figure 4). The results indicated that no obvious change was observed for the effective diameter and Zeta potential of the Ags-APMSNs over a wide range of pH from 3 to 9 and temperatures from 20 to 80°C.

### Peroxidase-Like Activity of Ags-APMSNs

**Figure 5:**
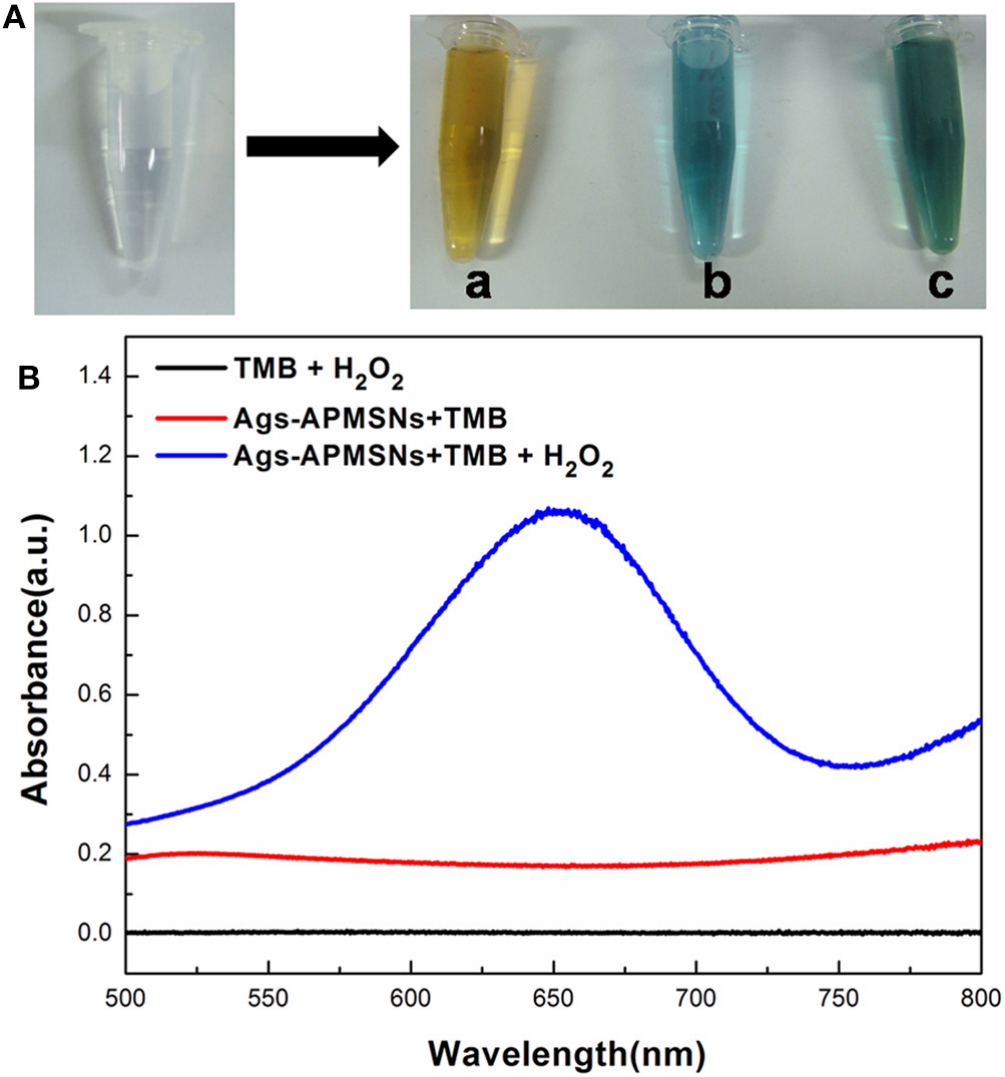
**(A)** Photographs of the reaction solutions in the absence and presence of Ags-APMSNs [**(a)** OPD, **(b)** TMB and **(c)** ABTS]. **(B)** UV-vis absorption spectra of the TMB-H_2_O_2_ (black line), TMB-Ags-APMSNs (red line), and TMB-H_2_O_2_-Ags-APMSNs (blue line) solution. The concentration of TMB was 0.4 mM, the concentration of H_2_O_2_ was 100 mM, the concentration of Ags-APMSNs was 0.05 nM and the reaction time was 10 min.

Previously, we found that Au@Pt NRs have intrinsic peroxidase-like activities, and the experimental and calculated results suggest the peroxidase-like activities of Au@Pt NRs can be ascribed to the larger surface exposure of the Pt island shell (Shen et al., 2015). Figure 5A showed the comparison of Ags-APMSN catalyzed oxidation reaction and color changes occurring with horseradish peroxidase (HRP) labels with different chromogens, such as TMB, OPD, and ABTS in the presence of substrate (H_2_O_2_), suggesting that the functionalized nanoprobe, Ags-APMSNs, had the peroxidase-like activity of Au@Pt NRs, consistent with the previous study (Liu et al., 2012).

Furthermore, we selected TMB as the colorimetric substrate to demonstrate this clearly. It has been well-established that TMB can be oxidized by hydrogen peroxide to form a blue color product, as judged by the appearance of the characteristic absorption peak at 650 nm. As shown in Figure 5B, in the absence of Ags-APMSNs, the TMB–H_2_O_2_ solution presented a negligible absorption in the range from 500 to 800 nm. In contrast, after addition of Ags-APMSNs, the solutions exhibited adsorption peaks centered at 650 nm. The significant increase in absorbance at 650 nm suggests that Ags-APMSNs catalyzes the oxidation of TMB by H_2_O_2_. All these observations confirmed the intrinsic peroxidase-like activity of the Ags-APMSNs, similar to that found in Au@Pt NRs previously (Liu et al., 2012).

On the other hand, for most nanozyme, the catalytic sites and recognition sites are not spatially separated. Hence, antigens-occupied catalytic sites should influence the catalytic activity of Au@Pt NRs and cause extra waste of catalysts. The encapsulation of Au@Pt NR in mesoporous SiO_2_ shell was able to hinder the interaction between nanoparticles and antigen moleculars, and the reacting substrates can directly access the Au@Pt NR cores through the mesopores within the SiO_2_ shells and the product can readily exit through these mesopores.

**Figure 6:**
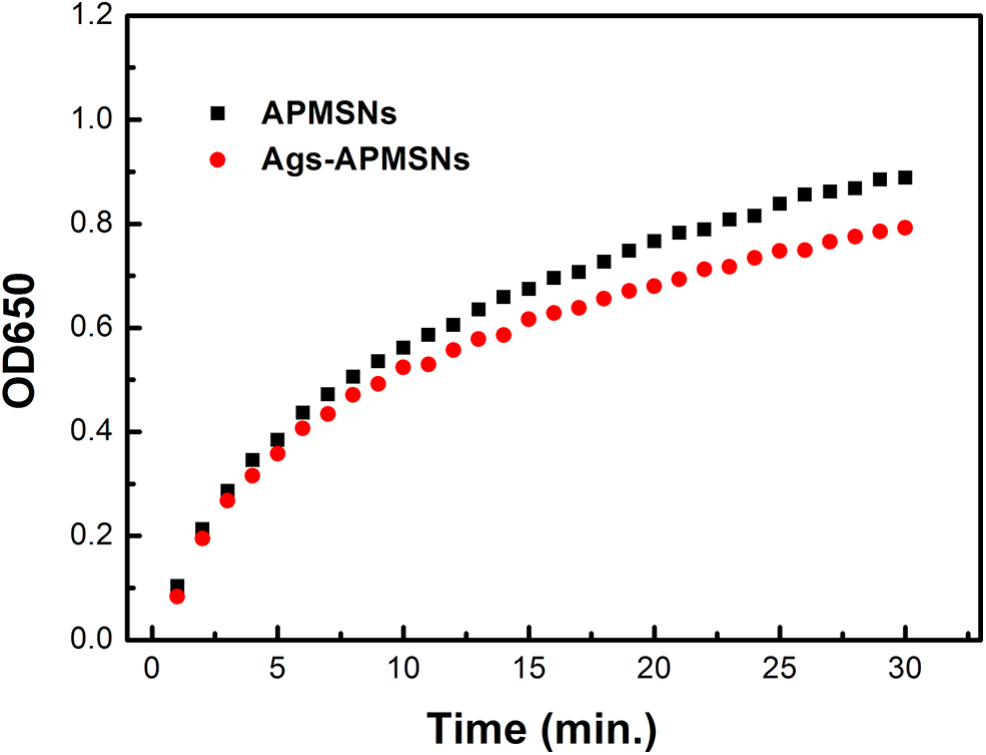
Absorbance evolution at 650 nm as a function over time for TMB oxidation in the presence of H_2_O_2_ catalyzed by APMSNs and Ags-APMSNs. The concentration of TMB was 0.4 mM, the concentration of H_2_O_2_ was 100 mM and the concentration of APMSNs or Ags-APMSNs was 0.025 nM.

As shown in Figure 6, the Ags-APMSNs exhibited an catalytic activity toward TMB in the presence of H_2_O_2_ similar to the case of APMSNs (the Ags-APMSNs maintained 90% activity of APMSNs). Since antigens conjugation had negligible influence on the catalytic activity of the APMSNs, this designed nanozyme have realized spatial separation of recognition sites and catalytic sites.

### Effect of Substrate and Ags-APMSNs Concentrations, pH, and Temperature

**Figure 7:**
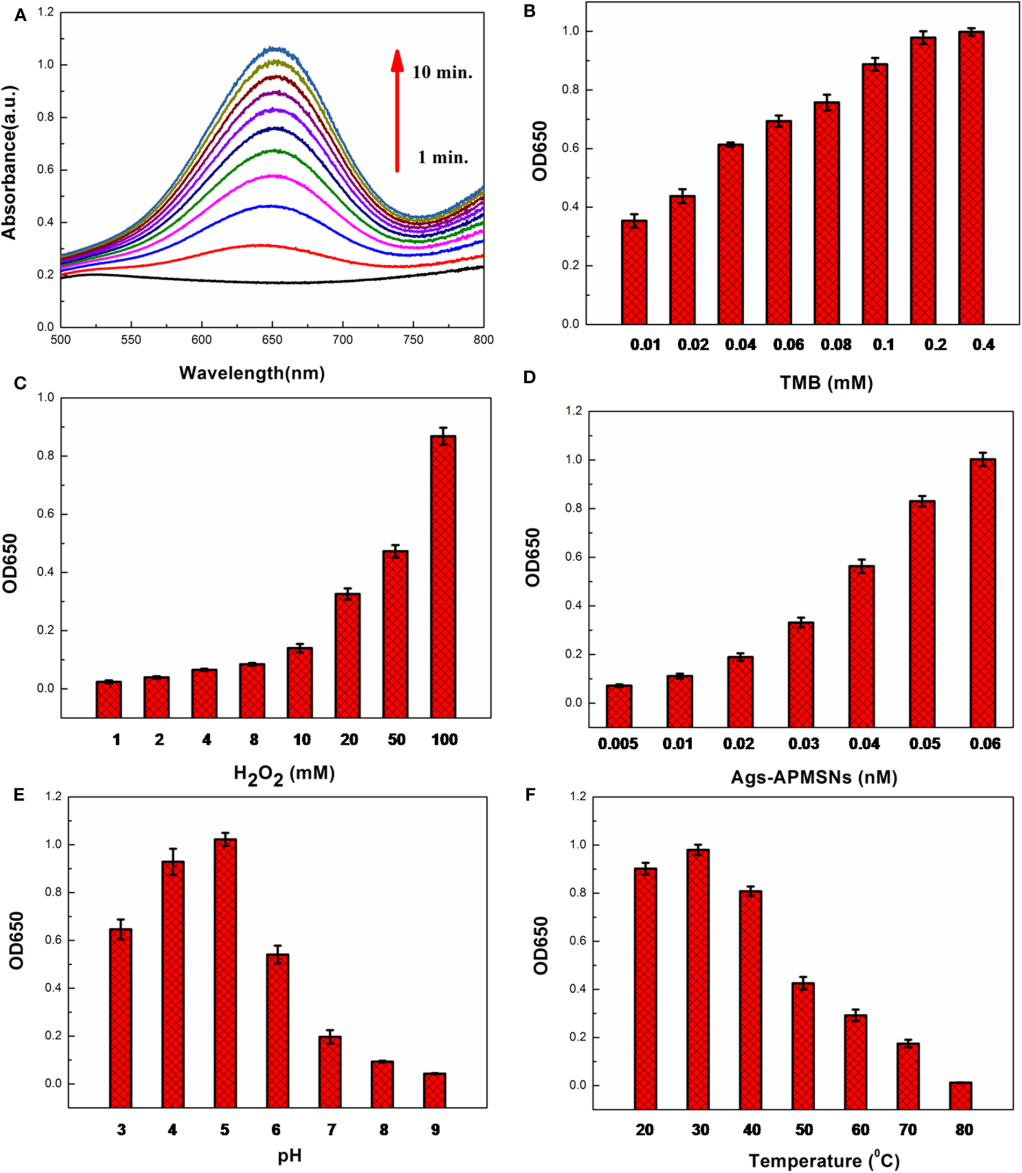
**(A)** Time-dependent absorbance changes of oxidation of TMB in the presence of H_2_O_2_. Effects of TMB concentration **(B)**, H_2_O_2_concentration **(C)**, Ags-APMSNs concentration **(D)**, pH **(E)**, and temperature **(F)** on catalytic activity of the Ags-APMSNs. Reaction conditions: **(A)** 0.4 mM TMB, 100 mM H_2_O_2_, and 0.05 nM APMSNs; **(B)** 100 mM H_2_O_2_, 0.05 nM APMSNs and 10 min; **(C)** 0.4 mM TMB, 0.05 nM APMSNs and 10 min; **(D)** 0.4 mM TMB, 100 mM H_2_O_2_ and 10 min; **(E)** 0.4 mM TMB, 100 mM H_2_O_2_, 0.05 nM APMSNs and 10 min at different pH; **(F)** 0.4 mM TMB, 100 mM H_2_O_2_, 0.05 nM APMSNs and 10 min at different temperatures.

It has been reported that the peroxidise-like activities activity of nanozyme is dependent on the substrate concentrations, pH of the reaction buffer and incubation temperature. The color reaction of TMB by H_2_O_2_ was employed to optimize the catalytic condition (Figure 7A). Figure 7B showed the dependence of absorbance at 650 nm on the concentration of TMB. The results indicated that the catalytic activity of Ags-APMSNs was rapidly increased initially with the increase of TMB concentration and then tended to saturate beyond 0.2 mM. Only slight changes in light intensity were observed when TMB concentration was above 0.2 mM. The influence of H_2_O_2_ concentration was investigated and the results were shown in Figure 7C. It can be seen that the activity of Ags-APMSNs increased with increased H_2_O_2_ concentration from 1 to 100 mM. As shown in Figure 7D, the catalytic activity of Ags-APMSNs increased gradually with the concentration of Ags-APMSNs from 0.005 to 0.06 nM. To know whether the catalytic activity of the Ags-APMSNs was dependent on pH of the reaction buffer and incubation temperature, the experiments were performed by varying the pH from 3 to 9 and temperatures from 20 to 80°C. The result suggested that the optimal pH is 5 and the optimal temperature is 30°C (Figures 7E,F), which was consistent with the feature of HRP labels.

Based on abovementioned results, the optimum conditions selected for the following biomedical assay were as follows: 0.4 mM TMB, 100 mM H_2_O_2_, pH 5, and 30°C.

### Application of Immunoassay

Figure 1B is a schematic of the whole assay steps used in this work, which the Ags-APMSNs were utilized as a nanoprobe instead of HRP labels for the determination of mumps-specific IgM antibodies. The assay was performed in mumps antigen-immobilized microplate wells. First, the samples were added and mumps-specific IgM antibodies present in the sera would bind to the antigens. After washing, bound IgM antibodies were detected using Ags-APMSNs, following which a detector system with chromogen substrate revealed the presence or absence of mumps-specific IgM antibodies in the test samples.

**Figure 8:**
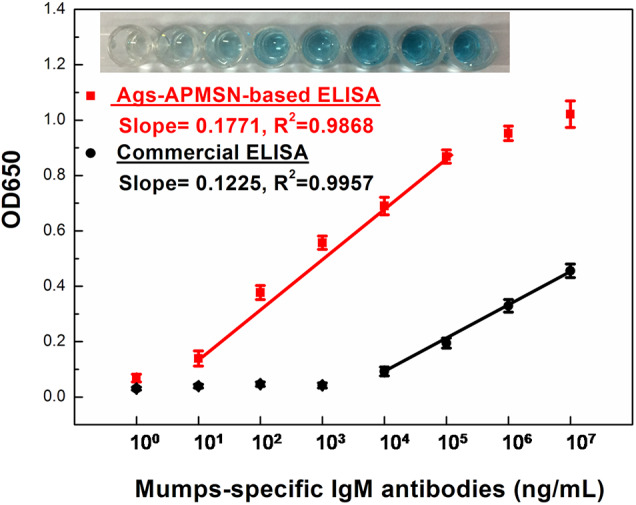
Calibration plot of absorption values at 650 nm vs. mumps-specific IgM antibodies at different concentration. The insets are the typical photograph of the wells of the Ags-APMSN-based ELISA.

Figure 8 showed the typical curve for different concentrations of mumps-specific IgM antibodies standard solutions. Owing to the high catalysis activity of Ags-APMSN, this proposed ELISA generated higher absorbance at 650 nm than the commercial ELISA and was able to quantify the target antigen faster. Significantly, compared to the commercial ELISA or captured-ELISA method (Glikmann et al., 1986), greatly amplified sensitivity was achieved as this proposed method has a limit of detection for mumps-specific IgM antibodies of 10 ng/mL in the linear range from 10 to 10^5^ ng/mL. Moreover, as the SiO_2_ shell have less interaction with plate surface, the unbound Ags-APMSN could be removed when the plates were washed. Thus, this proposed ELISA could reduce the high background signal or false-positive reactions as well.

**Figure 9:**
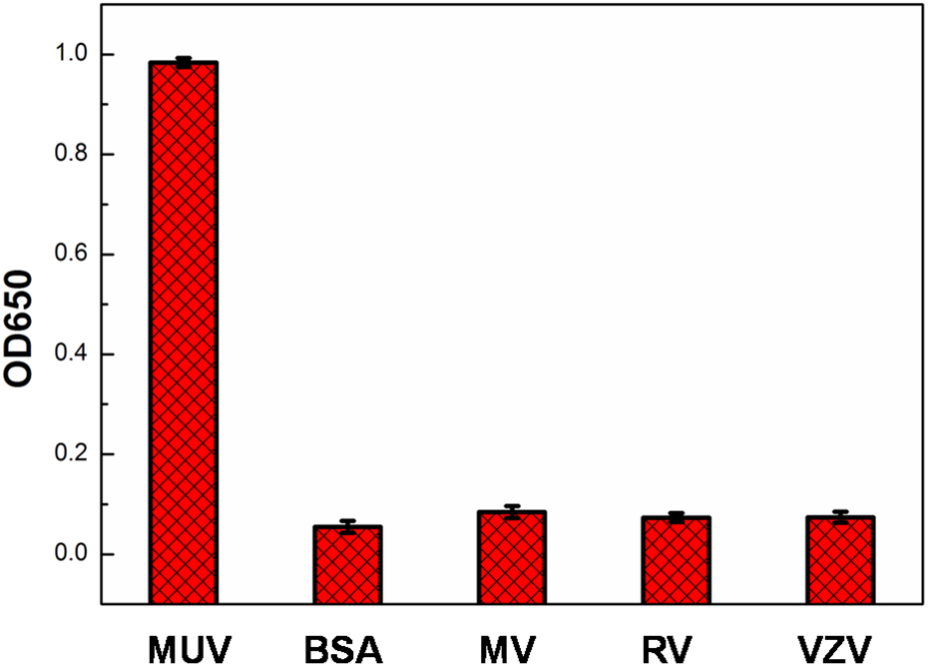
Specificity of mumps virus, BSA, measles virus (MV), rubella virus (RV), and varicella-zoster virus (VZV) positive serum using Ags-APMSN-based ELISA.

For testing if the detection of mumps-specific IgM antibodies was specific, control experiments were taken using BSA, measles virus, rubella virus and varicella-zoster virus positive serum. The selectivity of this proposed method was shown in Figure 9. In comparison to the mumps virus positive serum, there were no remarkable response in the other samples, indicating this proposed ELISA exhibited high selectivity toward mumps-specific IgM antibodies (Figure 9).

Also, the proposed ELISA based on Ags-APMSN catalyzed colorimetric immunoassay was tested in real blood serum for the diagnosis of mumps. The serum sample was obtained from Zaozhuang Municipal Center for Disease Control and Prevention and diluted 100 times in dilution buffer before assay (Figure S1 and Table S1). The results were compared with those obtained by the commercial ELISA (Table 2). As can be seen, the results obtained from this proposed ELISA agreed well with those obtained from the commercial ELISA. Therefore, the proposed method is suitable and satisfactory for diagnosis of mumps of real samples in clinical application.

**Table 2:** Comparison of assay performance of Ags-APMSN-based ELISA and commercial ELISA for real blood serum samples.

**Assay**	**Total**	**Positive**	**Negative**
Ags-APMSN-based ELISA	40	20	20
Commercial ELISA	40	20	20

## Conclusion

In conclusion, a novel nanoprobe was designed and synthesized. The results have shown that this functionalized nanoprobe, Ags-APMSNs, had intrinsic peroxidase-like activity, which can also serve as a medical diagnosis regent. In contrast to the natural enzyme labels, the obtained Ags-APMSNs are readily prepared, robust in harsh chemical environment, and cost-effective. Notably, the mesoporous SiO_2_ shell was able to hinder the interaction between catalytical nanoparticles and recognition antigens, retaining the catalytic activity of the inner active nanoparticle core. To facilitate further applications of this nanoprobe, an immunoassay was performed based on their enhanced catalytic activity. The nanoprobes were successfully utilized to detect the mumps-specific IgM antibodies in sera sensitively with the limit of detection as low as 10 ng/mL. The present work confirmed that the Ags-APMSNs were expected as a novel immunological probe for a wide range of practical applications in various areas, ranging from biosensing to clinical virus diagnosis.

## Data Availability Statement

The original contributions presented in the study are included in the article/Supplementary Material, further inquiries can be directed to the corresponding authors.

## Author Contributions

LL participated in the experiment and drew the scheme and figures. RC performed the experiments. JL wrote the paper with support from XW.

## Conflict of Interest

The authors declare that the research was conducted in the absence of any commercial or financial relationships that could be construed as a potential conflict of interest.
