# Supplementary material for: A Novel Nanoprobe Based on Core–Shell Au@Pt@Mesoporous SiO2 Nanozyme With Enhanced Activity and Stability for Mumps Virus Diagnosis
Source: Front Chem. 2020 Jun 5;8:463. doi: 10.3389/fchem.2020.00463 (PMC7290063; doi:10.3389/fchem.2020.00463)
Supplement: Supplementary file 1 [file Data_Sheet_1.PDF]

Additional files

A novel nanoprobe based on core–shell Au@Pt@mesoporous SiO_2 nanozyme with enhanced activity and stability for mumps virus diagnosis

Lin Long[1, 2†], Rui Cai[3, 4†], Jianbo Liu[1*] and Xiaochun Wu[3, 4*]

[1]College of Opto-electronic Engineering, Zaozhuang University, Zaozhuang, China

[2]Zaozhuang Municipal Center for Disease Control and Prevention, Zaozhuang, China

[3]CAS Key Laboratory of Standardization and Measurement for Nanotechnology, National Center for Nanoscience and Technology, Beijing, China

[4]University of Chinese Academy of Sciences, Beijing, China

[†]These authors contributed equally to this work
** Corresponding author email: linyibm@163.com (J. Liu), wuxc@nanoctr.cn (X. Wu))*

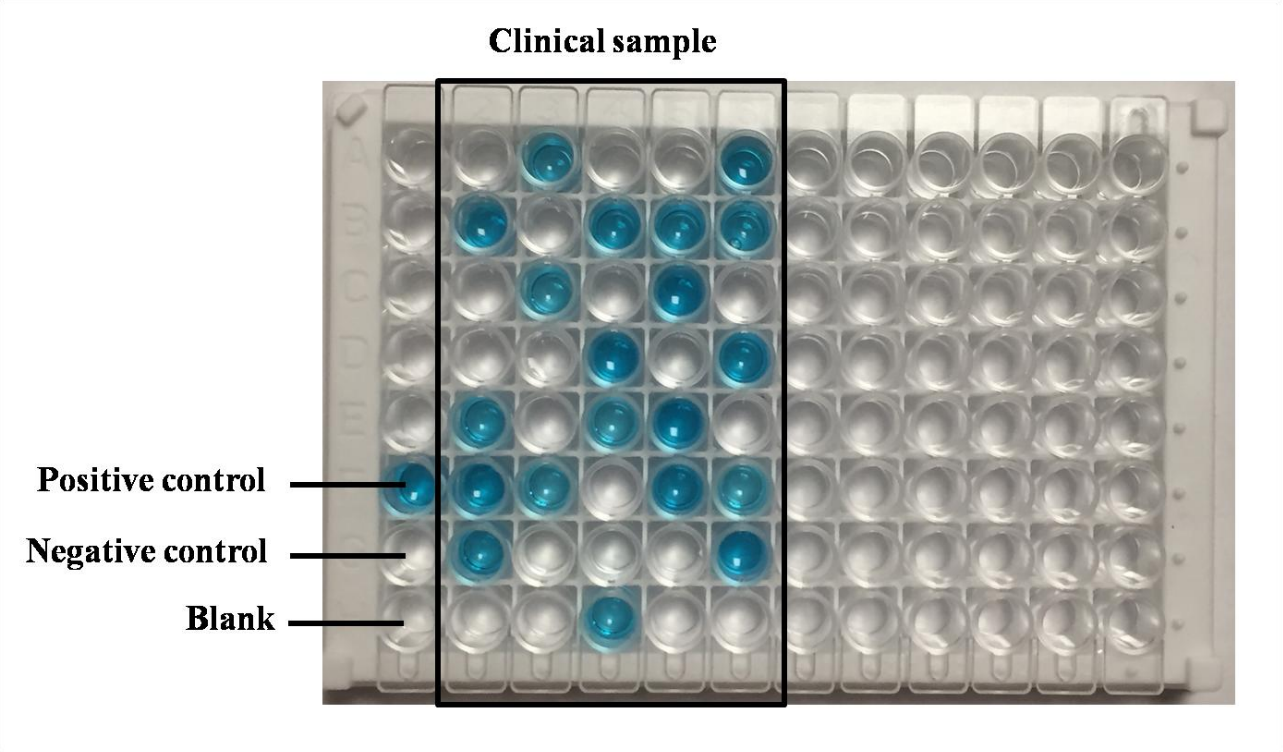

Figure S1 Colorimetric ELISA detection of mumps IgM in the clinical serum with Ags-APMSN-based ELISA

Table S1 Detection of mumps IgM in the clinical serum obtained by the Ags-APMSN-based ELISA.

Sample	OD650	Result (Positive / Negative)
1	1.119	Positive
2	1.084	Positive
3	0.894	Positive
4	1.09	Positive
5	0.936	Positive
6	1.129	Positive
7	0.961	Positive
8	1.113	Positive
9	1.108	Positive
10	0.978	Positive
11	0.985	Positive
12	0.996	Positive
13	1.207	Positive
14	0.936	Positive
15	1.012	Positive
16	1.121	Positive
17	0.986	Positive
18	0.968	Positive
19	0.913	Positive
20	0.952	Positive
21	0.102	Negative
22	0.173	Negative
23	0.142	Negative
24	0.165	Negative
25	0.103	Negative
26	0.144	Negative
27	0.122	Negative
28	0.134	Negative
29	0.152	Negative
30	0.173	Negative
31	0.145	Negative
32	0.137	Negative
33	0.125	Negative
34	0.132	Negative
35	0.165	Negative
36	0.0987	Negative
37	0.123	Negative
38	0.0846	Negative
39	0.173	Negative
40	0.104	Negative
